# Supplementary material for: Content-rich biological network constructed by mining PubMed abstracts
Source: BMC Bioinformatics. 2004 Oct 8;5:147. doi: 10.1186/1471-2105-5-147 (PMC528731; doi:10.1186/1471-2105-5-147)
Supplement: Additional File 2 — The original results of the above study (non-essential files are deleted to keep the file size under the limit set by BMC bioinformatics). [file 1471-2105-5-147-S2.bz2 › chilibotAdditionalFile2/dip50/600ID10092459E10979/html/PHES_PHET.html]

 


 **PHES** and **PHET** 
  
Found 54 abstracts in PubMed, retrieved 50.  
 

 What does Google say? 
 PDF only 
| .edu only 

---

**Interactive relationship** (e.g. stimulation, inhibition, etc)

**Neutral relationship**- Although its melting profile suggested a loose higher order structure, presumably influenced by the apparent loss of D loop T loop interaction necessary for forming a rigid L shaped tertiary structure, its aminoacylation capacity catalyzed by mt phenylalanyl tRNA synthetase PheRS  [ **PHET** ]   [ **PHES** ]  was nearly equal to that of Escherichia coli tRNAPhe.  Ref: 2473985 J Biol Chem, 1989
- The conformation of PheOH AMP and the network of interactions of its AMP moiety with PheRS  [ **PHET** ]   [ **PHES** ]  are reminiscent of the other class II synthetases.  Ref: 10092459 J Mol Biol, 1999
- The recognition of Phe by PheRS  [ **PHET** ]   [ **PHES** ]  is achieved through a mixture of multiple van der Waals interactions and hydrogen bonds.  Ref: 10092459 J Mol Biol, 1999
- The  **pheS**  gene corresponds to 1029 bp and the  **pheT**  gene to 2412 bp.  Ref: 2127701 Biochimie, 1990
- With the availability of the three dimensional structure of the complex between multisubunit PheRS  [ **PHET** ]   [ **PHES** ]  and tRNAPhe, a fuller picture of the specific tRNA aaRS interactions is beginning to emerge.  Ref: 9016717 Structure, 1997
- The interactions of PheRS  [ **PHET** ]   [ **PHES** ]  with tRNA stabilize the flexible N terminal part of the alpha subunit, which appeared to form the enzyme s 11th domain, comprising a coiled coil structure helical arm built up of two long antiparallel alpha helices.  Ref: 9016717 Structure, 1997
- It reveals that one tRNAPhe molecule binds across all four PheRS  [ **PHET** ]   [ **PHES** ]  subunits.  Ref: 9016717 Structure, 1997
- CONCLUSIONS The mode of interactions with tRNA explains the absolute necessity for the alphabeta 2 architecture of PheRS  [ **PHET** ]   [ **PHES** ] .  Ref: 9016717 Structure, 1997
- The interactions of tRNAPhe with PheRS  [ **PHET** ]   [ **PHES** ]  and particularly with the coiled coil domain of the alpha subunit result in conformational changes in TPsiC and D loops seen by comparison with uncomplexed yeast tRNAPhe.  Ref: 9016717 Structure, 1997
- In prokaryotes, the alpha and beta subunits of pheRS  [ **PHET** ]   [ **PHES** ]  are encoded by the genes  **pheS**  and  **pheT** , respectively.  Ref: 11281723 Protein Expr Purif, 2001
- The mutant  **pheS**  gene encoding the PheRS  [ **PHET** ]   [ **PHES** ]  alpha subunit was cloned and sequenced, and the deviation from the wild type gene was found to result in a Gly191 to Asp191 exchange.  Ref: 1959653 FEBS Lett, 1991
- Tn5luxAB was localized to a fragment of chromosomal DNA encoding the thrS, infC, rpmI, rplT,  **pheS**  and  **pheT**  genes, with Tn5 inserted in the 3 end of  **pheS** .  Ref: 12583892 FEMS Microbiol Lett, 2003
- Homogeneous PheRS  [ **PHET** ]   [ **PHES** ]  was purified from Bacillus subtilis and from E. coli transformed with the PheRS  [ **PHET** ]   [ **PHES** ]  gene.  Ref: 8325392 FEBS Lett, 1993
- A sulfate ion, which was identified on the protein surface, may mediate the interactions of PheRS  [ **PHET** ]   [ **PHES** ]  with DNA.  Ref: 11679717 Acta Crystallogr D Biol Crystallogr, 2001
- Both genes we named them  **pheS**  and  **pheT** , analogous to the corresponding genes of E. coli are carried by a 6.6 kilobase pair PstI fragment which also complements E. coli  **pheT**  mutations.  Ref: 2492510 J Bacteriol, 1989
- Secondly, mutations affecting either structural gene  **pheS**  or  **pheT**  caused a reduction in the mRNA levels for both  **pheS**  and  **pheT** .  Ref: 6215395 J Bacteriol, 1982
- Our results indicate that while OA may be capable of weakly inhibiting PheRS  [ **PHET** ]   [ **PHES** ] , the OA PheRS  [ **PHET** ]   [ **PHES** ]  complex cannot adopt the same conformation as does FAMP PheRS  [ **PHET** ]   [ **PHES** ] , contrary to previous assumptions.  Ref: 10447951 J Med Chem, 1999

**Non-interactive relationship** (e.g. studied together, co-existance, homology, etc.)

- The engineered PheRS  [ **PHET** ]   [ **PHES** ]  has 16 N terminal amino acids from beta galactosidase fused to the beta subunit.  Ref: 2336390 Nucleic Acids Res, 1990
- Unlike the catalytic alpha subunit, the beta subunit of heterodimeric alphabeta 2 phenylalanyl tRNA synthetase PheRS  [ **PHET** ]   [ **PHES** ]  has no invariant functional amino acids directly involved in the aminoacylation process as it is evident from the crystal structure of the T. thermophilus enzyme complexed with tRNAPhe.  Ref: 10049785 Biochem Biophys Res Commun, 1999
- The h mt TrpRS shows kinetic properties similar to human mitochondrial phenylalanyl tRNA synthetase h mt PheRS  [ **PHET** ]   [ **PHES** ] , and h mt TrpRS is not induced by interferon gamma as is hTrpRS.  Ref: 10828066 J Biol Chem, 2000
- Thus a compromise appears to exist between amino acid activation and tRNA charging, because slowing down the first step increases the rate of the second step, possibly as a result of decreased stability of the PheRS  [ **PHET** ]   [ **PHES** ] .amino acid AMP complex.  Ref: 7843418 FEBS Lett, 1995
- We describe the high level overexpression and purification of recombinant S. aureus pheRS  [ **PHET** ]   [ **PHES** ]  using  **pheS**  and  **pheT**  genes as part of an artificial operon in Escherichia coli.  Ref: 11281723 Protein Expr Purif, 2001
- Two ORFs with similarity to non archaeal PheRSs alpha subunits had previously been found in the genome sequence, but these results show that only one of them, MT742, is part of the active PheRS  [ **PHET** ]   [ **PHES** ] .  Ref: 10575359 Biochimie, 1999
- The alpha 2 beta 2 enzymes exhibit similarities to PheRS  [ **PHET** ]   [ **PHES** ]  also an alpha 2 beta 2 enzyme.  Ref: 8839980 Biol Chem Hoppe Seyler, 1996
- Since OTA is a phenylalanine analogue, this effect could be due to inhibition of phenylalanine tRNA synthetase PheRS  [ **PHET** ]   [ **PHES** ]  by competition of this mycotoxin with the amino acid.  Ref: 8325392 FEBS Lett, 1993
- The interaction of Thermus thermophilus phenylalanyl tRNA synthetase PheRS  [ **PHET** ]   [ **PHES** ]  with the 3 ; terminal nucleotide of tRNAPhe has been studied by affinity labeling to solve the problem arising from X ray crystallographic study the binding sites of phenylalanine and the 3 ; terminal nucleotide base were revealed to be identical in the crystal structures of PheRS  [ **PHET** ]   [ **PHES** ]  complexed with the substrates.  Ref: 11092959 Biochemistry Mosc , 2000
- The level of PheRS  [ **PHET** ]   [ **PHES** ]  alpha subunit like mRNA is regulated during differentiation but not during cell cycle progression.  Ref: 10375616 Gene, 1999
- The complex structures suggest that the PheRS  [ **PHET** ]   [ **PHES** ]  beta subunit may affect the first step of the reaction formation of phenylalanyl adenylate through the metal mediated conserved alpha beta subunit interface.  Ref: 10092459 J Mol Biol, 1999
- For comparative analysis we also report a procedure for the purification of native pheRS  [ **PHET** ]   [ **PHES** ]  from S. aureus Oxford Strain and demonstrate that Michaelis Menten parameters for both recombinant and native enzyme, at least for phenylalanine tRNA aminoacylation are comparable.  Ref: 11281723 Protein Expr Purif, 2001
- Coexpression of the mutant PheRS  [ **PHET** ]   [ **PHES** ]  and DHFR in a phenylalanine auxotrophic Escherichia coli host strain grown in p Br phe supplemented minimal medium resulted in 88% replacement of phenylalanine residues by p Br phe.  Ref: 10664452 FEBS Lett, 2000
- The modeling of tyrosine in the active site of PheRS  [ **PHET** ]   [ **PHES** ]  revealed no apparent close contacts between tyrosine and the PheRS  [ **PHET** ]   [ **PHES** ]  residues.  Ref: 10092459 J Mol Biol, 1999
- Crystals of native PheRS  [ **PHET** ]   [ **PHES** ]  were soaked in a solution containing phenylalanine and ATP in the presence of Mn 2 ions.  Ref: 11679717 Acta Crystallogr D Biol Crystallogr, 2001
- The dissociation constants for the eEF1A.GDP.tRNA and eEF1A.GDP.tRNAPhe.PheRS  [ **PHET** ]   [ **PHES** ]  complexes were found to be 20 nm and 9 nm, respectively.  Ref: 12354112 Eur J Biochem, 2002
- The nucleotide sequences of  **pheS**  and of the beginning of  **pheT**  have been determined.  Ref: 6317865 J Mol Biol, 1983
- Functional activity of the covalent complex of PheRS  [ **PHET** ]   [ **PHES** ]  with the s4U monosubstituted transcript has been shown by aminoacylation of 60% of the enzyme cross linked tRNA.  Ref: 9613588 FEBS Lett, 1998
- This, in turn, suggests that the previously observed antagonistic effects of aspartame and piroxicam are more likely due to their prevention of OA binding to human serum albumin than to PheRS  [ **PHET** ]   [ **PHES** ] , which is in agreement with binding studies as well as with preliminary simulations performed in our laboratory.  Ref: 10447951 J Med Chem, 1999
- These three latter aaRS share three new sequence motifs with AspRS, AsnRS, LysRS, HisRS and the beta subunit of PheRS  [ **PHET** ]   [ **PHES** ] .  Ref: 2203971 Nature, 1990
- Plasmid pB1 carries the three genes thrS,  **pheS** , and  **pheT** , and strains carrying the plasmid show enhanced levels of mRNA corresponding to these genes.  Ref: 6215395 J Bacteriol, 1982
- ... pre steady state analysis revealed higher rates of tRNA charging by both the A294S PheRS  [ **PHET** ]   [ **PHES** ]  phenylalanyl adenylate and the A294G PheRS  [ **PHET** ]   [ **PHES** ]  p Cl phenylalanyl adenylate.  Ref: 7843418 FEBS Lett, 1995
- Here we present the data on association of the eEF1A.GDP.tRNA complex with phenylalanyl tRNA synthetase PheRS  [ **PHET** ]   [ **PHES** ] , e.g. the formation of the above quaternary complex detected by the gel retardation and surface plasmon resonance techniques.  Ref: 12354112 Eur J Biochem, 2002
- This is the first report of the cloning of the full length cDNA for the murine PheRS  [ **PHET** ]   [ **PHES** ]  regulatory alpha subunit like protein.  Ref: 10375616 Gene, 1999
- They are compared to the available data concerning the primary structure of the subunits, as deduced from nucleotide sequencing of the  **pheS**   **pheT**  operon.  Ref: 6360212 Biochim Biophys Acta, 1983
- Phenylalanyl tRNA synthetase PheRS  [ **PHET** ]   [ **PHES** ] .  Ref: 1959653 FEBS Lett, 1991
- In particular, it is suggested that the monomeric PheRS  [ **PHET** ]   [ **PHES** ]  from the yeast mitochondrion is a chimera of the alpha and beta chains of the standard tetrameric protein.  Ref: 12572618 Mol Biol Evol, 1998
- The predicted amino acid sequence is 589 amino acids and shares 45% identity with the yeast cytoplasmic phenylalanyl tRNA synthetase PheRS  [ **PHET** ]   [ **PHES** ]  regulatory alpha subunit.  Ref: 10375616 Gene, 1999
- We also revealed a direct interaction of PheRS  [ **PHET** ]   [ **PHES** ]  with eEF1A in the absence of tRNAPhe Kd = 21 nm.  Ref: 12354112 Eur J Biochem, 2002
- The genes  **pheS**  and  **pheT**  code, respectively, for the small and large subunits of phenylalanyl tRNA synthetase, an alpha 2 beta 2 enzyme.  Ref: 6317865 J Mol Biol, 1983
- Seventeen different kinds of deletions extending into  **pheS**  of  **pheT**  were identified.  Ref: 7012115 J Bacteriol, 1981
- The possible exploitation of the Gly294 mutant PheRS  [ **PHET** ]   [ **PHES** ]  for the incorporation of non proteinogenic amino acids into proteins is discussed.  Ref: 1942071 J Mol Biol, 1991
- It is suggested that one of the terminators is an attenuation site controlling the extension of transcription into  **pheS**  and  **pheT** .  Ref: 6368838 J Mol Biol, 1984
- Although only limited similarities are found in the protein sequences deduced from their known gene sequences, the presence of common epitopes in GlyRS and PheRS  [ **PHET** ]   [ **PHES** ]  adds to a rather long list of physical and chemical similarities between those proteins.  Ref: 3284463 Arch Biochem Biophys, 1988
- The construction of three lambda bacteriophages containing parts of the structural gene for threonyl tRNA synthetase, thrS, and those for the two subunits of phenylalanyl tRNA synthetases,  **pheS**  and  **pheT** , is described.  Ref: 6215395 J Bacteriol, 1982
- Antibodies to Escherichia coli glycyl tRNA synthetase GlyRS cross react extensively with E. coli phenylalanyl tRNA synthetase PheRS  [ **PHET** ]   [ **PHES** ] .  Ref: 3284463 Arch Biochem Biophys, 1988
- Comparing the aminoacylation efficiencies of several combinations of tRNAPheS and PheRSs from various sources, including bovine mitochondria, bovine and yeast cytosols, E. coli, Thermus thermophilus, and Sulfolobus acidocaldarius, it was clarified that mt PheRS  [ **PHET** ]   [ **PHES** ]  was able to aminoacylate all the above mentioned tRNAPhe species, albeit with varying degrees of efficiency.  Ref: 2473985 J Biol Chem, 1989
- We thus propose that motif 3 participates in the formation of the Phe binding site of PheRS  [ **PHET** ]   [ **PHES** ] .  Ref: 1942071 J Mol Biol, 1991
- This result implies that the proofreading mechanism against activated tyrosine, rather than direct recognition, may play the major role in the PheRS  [ **PHET** ]   [ **PHES** ]  specificity.  Ref: 10092459 J Mol Biol, 1999
- Identification, cloning, and expression of a functional phenylalanyl tRNA synthetase pheRS  [ **PHET** ]   [ **PHES** ]  from Staphylococcus aureus.  Ref: 11281723 Protein Expr Purif, 2001
- Misaminoacylation was not observed for the mt tRNAPhe mt PheRS  [ **PHET** ]   [ **PHES** ]  system.  Ref: 2473985 J Biol Chem, 1989
- The 14C tRNA labeled chymotryptic peptides of PheRS  [ **PHET** ]   [ **PHES** ]  were purified by both gel filtration and reverse phase high performance liquid chromatography.  Ref: 2823880 Biochemistry, 1987
- This broad charging spectrum suggests that mt PheRS  [ **PHET** ]   [ **PHES** ]  possesses a relatively simple recognition mechanism toward its substrate, tRNAPhe.  Ref: 2473985 J Biol Chem, 1989
- Visible conformational changes were detected in the active site area adjacent to the position of the Phe AMP, compared with the structure of PheRS  [ **PHET** ]   [ **PHES** ]  complexed with a synthetic adenylate analogue phenylalaninyl adenylate.  Ref: 11679717 Acta Crystallogr D Biol Crystallogr, 2001
- Periodate oxidized tRNA Phe tRNA oxPhe behaves as a specific affinity label of tetrameric Escherichia coli phenylalanyl tRNA synthetase PheRS  [ **PHET** ]   [ **PHES** ] .  Ref: 2823880 Biochemistry, 1987
- Both Phe and PheOH AMP are engulfed in the active site cleft of the catalytic alpha subunit of PheRS  [ **PHET** ]   [ **PHES** ] , and neither makes contact with the PheRS  [ **PHET** ]   [ **PHES** ]  beta subunit.  Ref: 10092459 J Mol Biol, 1999
- HierDock identifies p fluorophenylalanine and 3 thienylalanine as especially good substrates for PheRS  [ **PHET** ]   [ **PHES** ] , in agreement with experiment.  Ref: 12452720 J Am Chem Soc, 2002
- The genes  **pheS**  and  **pheT**  encode the alpha and beta subunits with a molecular weight of 39 and 87 kD, respectively.  Ref: 1508711 Nucleic Acids Res, 1992
- The product of the FRS2 FRS1 operon is not able to complement thermosensitive E. coli PheRS  [ **PHET** ]   [ **PHES** ] , indicating the lack of heterologous aminoacylation in vivo.  Ref: 2336390 Nucleic Acids Res, 1990
- Four plasmids were isolated which complemented  **pheS**  and  **pheT**  thermosensitive mutations but which do not carry  **pheS**  or  **pheT** , the structural genes for phenylalanyl tRNA synthetase.  Ref: 6300764 Nucleic Acids Res, 1983
- The uniqueness of PheRS  [ **PHET** ]   [ **PHES** ]  in charging 2 OH of tRNA is dictated by the size of its adenine binding pocket and by the local conformation of the tRNA s CCA end.  Ref: 9016717 Structure, 1997
- The N terminal 314 amino acid residues appear to be analogous to the alpha subunit of the prokaryotic PheRS  [ **PHET** ]   [ **PHES** ] , while the C terminal 100 amino acid residues correspond to a region of the beta subunit known to interact with the anticodon of tRNAPhe.  Ref: 10329163 J Mol Biol, 1999
- The highest affinity of T. thermophilus PheRS  [ **PHET** ]   [ **PHES** ]  for cognate tRNA, observed for synthetase tRNA complexes, results in 100 3000 fold binding discrimination against non cognate tRNAs.  Ref: 12382236 J Mol Recognit< MedlineTA>J Mol Recognit, 1999
- Two of the R M systems, NmeBI and NmeDI, were located at homologous positions between the phenylalanyl tRNA synthetase genes  **pheS**  and  **pheT** , which appeared to be a preferential target for the insertion of foreign DNA in meningococci.  Ref: 10671450 J Bacteriol, 2000
- Comparisons with the sequences of PheRS  [ **PHET** ]   [ **PHES** ]  from yeast and Drosophila mitochondria indicate they are 42 % and 51 % identical with the human mtPheRS, respectively.  Ref: 10329163 J Mol Biol, 1999
- Firstly, a cis acting insertion located before the structural genes for phenylalanyl tRNA synthetase caused a major decrease in both  **pheS**  and  **pheT**  mRNA.  Ref: 6215395 J Bacteriol, 1982
- The correct positioning of the 3 ; terminal nucleotide of tRNAPhe corresponding to the structure of the productive complex with PheRS  [ **PHET** ]   [ **PHES** ]  is therefore promoted only in the presence of all three substrates.  Ref: 11092959 Biochemistry Mosc , 2000
- the gene order is thrS infC  **pheS**   **pheT** .  Ref: 6368838 J Mol Biol, 1984
- Detailed comparison between the structures of the Escherichia coli biotin synthetase repressor protein BirA and the recently solved Thermus thermophilus phenylalanyl tRNA synthetase PheRS  [ **PHET** ]   [ **PHES** ]  reveals significant similarities outside their respective catalytic domains.  Ref: 8563641 Protein Sci, 1995
- No promoter activity was found between  **pheS**  and  **pheT**  with either of the two techniques.  Ref: 6368838 J Mol Biol, 1984
- Evolutionary origins of this small monomeric human mtPheRS are unknown implications are that this enzyme is a result of the simplification of the more complex alphabeta 2 bacterial PheRS  [ **PHET** ]   [ **PHES** ]  in which specific functional regions were retained.  Ref: 10329163 J Mol Biol, 1999
- In this study, we incorporate para bromophenylalanine p Br phe into a model target protein, mouse dihydrofolate reductase DHFR, by using a bacterial phenylalanyl tRNA synthetase PheRS  [ **PHET** ]   [ **PHES** ]  variant with relaxed substrate specificity.  Ref: 10664452 FEBS Lett, 2000
- In addition, antibodies directed at the alpha and beta subunits of GlyRS inhibit both GlyRS and PheRS  [ **PHET** ]   [ **PHES** ]  in the same relative manner, indicating that the function as well as the structure of subunits is similar in each enzyme.  Ref: 3284463 Arch Biochem Biophys, 1988
- The Km and Ki values of PheRS  [ **PHET** ]   [ **PHES** ] , respectively, for phenylalanine and OTA were measured and their concentrations within the cell determined.  Ref: 8325392 FEBS Lett, 1993
- RESULTS The crystal structure of Thermus thermophilus PheRS  [ **PHET** ]   [ **PHES** ]  complexed with cognate tRNA has been solved at 3.28 A resolution.  Ref: 9016717 Structure, 1997
- In this paper, we describe a computational protocol HierDock for predicting the relative energies of binding of phenylalanine analogues to phenylalanyl tRNA synthetase PheRS  [ **PHET** ]   [ **PHES** ] .  Ref: 12452720 J Am Chem Soc, 2002
- Read clockwise, the order is aroD, aroH,  **pheT** , and  **pheS** .  Ref: 3032903 J Bacteriol, 1987
- It was also shown that the 2 3 fold increase of PheRS  [ **PHET** ]   [ **PHES** ]  in OTA treated cells is not due to phenylalanine controlled attenuation regulation.  Ref: 8325392 FEBS Lett, 1993
- The structural similarity between PheRS  [ **PHET** ]   [ **PHES** ]  and histidyl tRNA synthetase extends to the amino acid binding site, which is normally unique for each enzyme.  Ref: 10092459 J Mol Biol, 1999
- It also shows that the two genes following infC, namely  **pheS**  and  **pheT** , which form the phenylalanyl tRNA synthetase operon, are not expressed from infC s promoter.  Ref: 7050631 Mol Gen Genet, 1982
- The transfer of amino acid to tRNA by Escherichia coli phenylalanyl tRNA synthetase PheRS  [ **PHET** ]   [ **PHES** ]  was studied using replacements of Ala294 in the alpha subunit previously shown to have modified amino acid specificity.  Ref: 7843418 FEBS Lett, 1995
- these genes included  **pheS**  and  **pheT** , which code for the alpha and beta subunits of phenylalanyl transfer ribonucleic acid synthetase, and thrS, the structural gene for threonyl transfer ribonucleic acid synthetase.  Ref: 7012115 J Bacteriol, 1981
- Escherichia coli threonyl tRNA synthetase EC 6.1.1.3 expression has been examined in an acellular protein synthesizing system programmed with a plasmid DNA carrying thrS, infC,  **pheS** , and  **pheT** , the gene for threonyl tRNA synthetase, initiation factor 3, and the two protomers of phenylalanyl tRNA synthetase EC 6.1.1.20, respectively.  Ref: 6325425 J Biol Chem, 1984
- Phenylalanyl tRNA synthetase EC 6.1.1.20 has been purified to homogeneity from a 100 fold overproducing Escherichia coli strain carrying a hybrid pBR322 plasmid containing the  **pheS**   **pheT**  locus.  Ref: 6360212 Biochim Biophys Acta, 1983
- The sequences reported here will greatly aid in the three dimensional structure determination of T. thermophilus PheRS  [ **PHET** ]   [ **PHES** ] , a heterotetrameric alpha 2 beta 2, class II aminoacyl tRNA synthetase.  Ref: 1451792 FEBS Lett, 1992
- Phenylalanyl tRNA synthetase pheRS  [ **PHET** ]   [ **PHES** ]  is unique among aminoacyl tRNA synthetases in that it is a heterotetrameric enzyme composed of two alpha subunits and two larger beta subunits.  Ref: 11281723 Protein Expr Purif, 2001
- Comparative analysis of the cross linked products in the absence and in the presence of small substrates revealed ATP and aminoacyl adenylate to effect the interaction of the tRNAPhe acceptor end with PheRS  [ **PHET** ]   [ **PHES** ] .  Ref: 11092959 Biochemistry Mosc , 2000
- Human phenylalanyl tRNA synthetase PheRS  [ **PHET** ]   [ **PHES** ]  was cloned and expressed in Escherichia coli.  Ref: 11858721 Protein Expr Purif, 2002
- FRS1 and FRS2, the structural genes encoding the large alpha and small beta subunits of yeast phenylalanyl tRNA synthetase PheRS  [ **PHET** ]   [ **PHES** ]  were placed under the control of the lacZ promoter by creating an artificial operon.  Ref: 2336390 Nucleic Acids Res, 1990
- While crystals suitable for X ray diffraction analyses are available of phenylalanyl tRNA synthetase PheRS  [ **PHET** ]   [ **PHES** ]  from the thermophilic bacterium Thermus thermophilus, neither the primary structure of its constituent alpha and beta subunits nor the nucleotide sequence of the corresponding  **pheS**  and  **pheT**  genes were known.  Ref: 1451792 FEBS Lett, 1992
- Mutants with deletions covering either  **pheS**  or  **pheT** , but not both, were analyzed further by assay of phenylalanyl transfer ribonucleic acid synthetase.  Ref: 7012115 J Bacteriol, 1981
- The loss is only 1.5 fold when tRNA Phe CC 3 deoxyadenosine is aminoacylated by yeast cytoplasmic PheRS  [ **PHET** ]   [ **PHES** ]  Sprinzl, M.  Ref: 8555164 Biochemistry, 1996
- Synthesis of mRNA in the acellular DNA dependent protein synthesizing system has been measured by molecular hybridization to gene specific lambda DNA probes corresponding to thrS,  **pheS** , and  **pheT** .  Ref: 6325425 J Biol Chem, 1984
- In this report we describe the isolation of a DNA fragment 3.52 kb containing the  **pheS**  and  **pheT**  genes from a Staphylococcus aureus WCUH29 genomic DNA library.  Ref: 11281723 Protein Expr Purif, 2001
- The data obtained, combined with kinetic data on aminoacylation, clearly show that PheRS  [ **PHET** ]   [ **PHES** ]  exhibits more tRNA selectivity at the level of binding than at the level of catalysis.  Ref: 12382236 J Mol Recognit< MedlineTA>J Mol Recognit, 2001
- The selected cDNA encodes a protein that is a close homolog of one subunit of prokaryote and yeast phenylalanyl tRNA synthetase PheRS  [ **PHET** ]   [ **PHES** ] .  Ref: 9177188 Proc Natl Acad Sci U S A, 1997
- Motif 2 may thus participate in the formation of the phenylalanine binding site in PheRS  [ **PHET** ]   [ **PHES** ] .  Ref: 1959653 FEBS Lett, 1991
- The extent of tRNA recognition at the level of binding by Thermus thermophilus phenylalanyl tRNA synthetase PheRS  [ **PHET** ]   [ **PHES** ] , one of the most complex class II synthetases, has been studied by independent measurements of the enzyme association with wild type and mutant tRNA Phe s as well as with non cognate tRNAs.  Ref: 12382236 J Mol Recognit< MedlineTA>J Mol Recognit, 1991
- Among other mechanisms of toxicity, it has been suggested that OA inhibits phenylalanyl tRNA synthetase PheRS  [ **PHET** ]   [ **PHES** ] , thereby reducing protein synthesis.  Ref: 10447951 J Med Chem, 1999
- The results suggest specific binding of the 3 ; terminal nucleotide of tRNAPhe by the catalytic subunit of PheRS  [ **PHET** ]   [ **PHES** ]  in the absence of other substrates.  Ref: 11092959 Biochemistry Mosc , 2000
- Starting with the crystal structure of Thermus thermophilus PheRS  [ **PHET** ]   [ **PHES** ]  without bound ligand, HierDock predicts the binding site of phenylalanine Phe within 1.1 A of that revealed by the crystal structure of PheRS  [ **PHET** ]   [ **PHES** ]  cocrystallized with Phe.  Ref: 12452720 J Am Chem Soc, 2002
- Detailed analysis indicates that unlike the alphabeta 2 structure of the prokaryotic and eukaryotic cytoplasmic forms of PheRS  [ **PHET** ]   [ **PHES** ] , the human mtPheRS consists of a single polypeptide chain.  Ref: 10329163 J Mol Biol, 1999
- Both analogs have been cross linked to Thermus thermophilus phenylalanyl tRNA synthetase PheRS  [ **PHET** ]   [ **PHES** ]  and the specificity of the cross linking has been demonstrated.  Ref: 9613588 FEBS Lett, 1998
- thermophilus PheRS  [ **PHET** ]   [ **PHES** ]  approaches the anticodon loop from the minor groove side.  Ref: 9016717 Structure, 1997
- We report here the genetic analysis of an Escherichia coli mutant strain which is auxotrophic for phenylalanine because it has a PheRS  [ **PHET** ]   [ **PHES** ]  with a decreased affinity for phenylalanine.  Ref: 1959653 FEBS Lett, 1991
- The alphabeta 2 phenylalanyl tRNA synthetase PheRS  [ **PHET** ]   [ **PHES** ]  is one of the most complex enzymes in the aaRS family and is characterized by anomalous charging properties.  Ref: 9016717 Structure, 1997
- In addition, PheRS  [ **PHET** ]   [ **PHES** ]  residues Trpalpha149, Seralpha180, Hisalpha178, Argalpha204, Glnalpha218, and Glualpha220 form a total of seven hydrogen bonds with the main chain atoms of Phe.  Ref: 10092459 J Mol Biol, 1999
- 1973 Nature 24 5, indicating mechanistic differences between the two PheRS  [ **PHET** ]   [ **PHES** ]  s active sites for the amino acid transfer step.  Ref: 8555164 Biochemistry, 1996
- The ochratoxin A OTA metabolite 4R 4 hydroxyochratoxin A 4R OTA inhibits the aminoacylation of phenylalanine tRNA catalyzed by phenylalanyl tRNA synthetase PheRS  [ **PHET** ]   [ **PHES** ]  with a Ki value of 0.9 mM as compared to 1.3 mM for OTA.  Ref: 6652810 Chem Biol Interact, 1983
- The phenylalanyl tRNA synthetase operon is composed of two adjacent, cotranscribed genes,  **pheS**  and  **pheT** , corresponding respectively to the small and large subunit of phenylalanyl tRNA synthetase.  Ref: 6426518 Biochim Biophys Acta, 1984
- In vivo and in vitro results demonstrated that Phe293 and Phe295 are not directly involved in substrate binding, but replacements of those residues affected PheRS  [ **PHET** ]   [ **PHES** ]  stability.  Ref: 1942071 J Mol Biol, 1991
- This does not suggest PheRS  [ **PHET** ]   [ **PHES** ]  to be the target of OTA in cell growth and protein synthesis inhibition in Bacillus subtilis.  Ref: 8325392 FEBS Lett, 1993
- These deletions unambiguously defined the gene order as aroD pps himA  **pheT**   **pheS**  thrS pfkB.  Ref: 7012115 J Bacteriol, 1981
- In an attempt to detect the phenylalanine Phe binding site, two Escherichia coli PheRS  [ **PHET** ]   [ **PHES** ]  mutant strains  **pheS** , which were resistant to p fluorophenylalanine p F Phe were analysed genetically.  Ref: 1942071 J Mol Biol, 1991
- Of particular interest was the Gly294 PheRS  [ **PHET** ]   [ **PHES** ]  in which presumably an enlarged cavity for the para position of the aromatic ring allowed an increased aminoacylation of tRNA with p F Phe.  Ref: 1942071 J Mol Biol, 1991
- A few backbone mediated contacts of PheRS  [ **PHET** ]   [ **PHES** ]  with the acceptor and anticodon stems revealed in the crystal structure do not contribute to tRNA Phe discrimination, their role being limited to stabilization of the complex.  Ref: 12382236 J Mol Recognit< MedlineTA>J Mol Recognit, 1991
- The calculated binding energies of Phe analogues in PheRS  [ **PHET** ]   [ **PHES** ] , using HierDock, correlate well with the translational activities of the same analogues in Escherichia coli.  Ref: 12452720 J Am Chem Soc, 2002
- Aminoacylation activity of the overproduced human PheRS  [ **PHET** ]   [ **PHES** ]  in the crude bacterial extract is two orders of magnitude higher than the corresponding activity in human placenta and the yield of the recombinant enzyme overproduced in E. coli is five times higher.  Ref: 11858721 Protein Expr Purif, 2002
- Using consensus sequences derived from conserved regions of the alpha and beta subunits from bacterial PheRS  [ **PHET** ]   [ **PHES** ] , two partially sequenced cDNA clones were identified.  Ref: 10329163 J Mol Biol, 1999
- Remarkably, both rates of aminoacyl transfer and amino acid specificities are greater for RNA 77 than measured for protein PheRS  [ **PHET** ]   [ **PHES** ] .  Ref: 10318907 Proc Natl Acad Sci U S A, 1999
- Neither the tertiary structure nor the location of active sites are known for phenylalanyl tRNA synthetase PheRS  [ **PHET** ]   [ **PHES** ] .  Ref: 1942071 J Mol Biol, 1991
- These include the gyrA gyrB dnaA dnaN gene cluster, the rRNA gene cluster, fla, flgE, groEL, recA, the rho hip cluster, the dnaK dnaJ grpE cluster, the  **pheT**   **pheS**  cluster, and the genes which encode the potent immunogen proteins p22A, p39 and p83.  Ref: 8102774 Mol Microbiol, 1993
- The latter produced about 40 times more PheRS  [ **PHET** ]   [ **PHES** ]  than B. subtilis.  Ref: 8325392 FEBS Lett, 1993
- We cloned, sequenced, and expressed human PheRS  [ **PHET** ]   [ **PHES** ] .  Ref: 10049785 Biochem Biophys Res Commun, 1999
- The crystal structure of phenylalanyl tRNA synthetase PheRS  [ **PHET** ]   [ **PHES** ]  from Thermus thermophilus, a class II aminoacyl tRNA synthetase, complexed with phenylalanyl adenylate Phe AMP was determined at 2.6 A resolution.  Ref: 11679717 Acta Crystallogr D Biol Crystallogr, 2001
- Using the crystal structure of PheRS  [ **PHET** ]   [ **PHES** ]  from Thermusthermophilus, we have modeled its interactions with OA as well as with phenylalanyl adenylate FAMP, the high affinity intermediate substrate of PheRS  [ **PHET** ]   [ **PHES** ] .  Ref: 10447951 J Med Chem, 1999
- Three different types of alphabeta 2 heterodimers of human PheRS  [ **PHET** ]   [ **PHES** ]  carrying HT at the N terminus of either of two alpha or beta subunits or simultaneously on both of them were overproduced and purified.  Ref: 11858721 Protein Expr Purif, 2002
- The alpha and beta subunits of phenylalanyl tRNA synthetase are encoded by the  **pheS**  and  **pheT**  genes, respectively.  Ref: 6368838 J Mol Biol, 1984
- The crystal structures of Thermus thermophilus phenylalanyl tRNA synthetase PheRS  [ **PHET** ]   [ **PHES** ]  complexed with phenylalanine and phenylalaninyl adenylate PheOH AMP, the synthetic analogue of phenylalanyl adenylate, have been determined at 2.7A and 2.5A resolution, respectively.  Ref: 10092459 J Mol Biol, 1999
- Sodium dodecyl sulfate polyacrylamide gel electrophoresis analysis of the PheRS  [ **PHET** ]   [ **PHES** ]  14C tRNA oxPhe covalent complex indicates that the large alpha, Mr 87K subunit of the enzyme interacts with the 3 adenosine of tRNA oxPhe.  Ref: 2823880 Biochemistry, 1987
